# Heat Stress Alters the Effect of *Eimeria maxima* Infection on Ileal Amino Acids Digestibility and Transporters Expression in Meat-Type Chickens

**DOI:** 10.3390/ani12121554

**Published:** 2022-06-16

**Authors:** Ahmed F. A. Ghareeb, Gustavo H. Schneiders, James C. Foutz, Marie C. Milfort, Alberta L. Fuller, Jianmin Yuan, Romdhane Rekaya, Samuel E. Aggrey

**Affiliations:** 1 Department of Poultry Science, University of Georgia, 110 Cedar St, Athens, GA 30602, USA; ahmed.ghareeb@uga.edu (A.F.A.G.); gustavo.schneiders@merck.com (G.H.S.); james.foutz@boehringer-ingelheim.com (J.C.F.); milfort@uga.edu (M.C.M.); alfuller@uga.edu (A.L.F.); 2 Merck Animal Health, 2 Giralda Farms, Madison, NJ 07940, USA; 3 Boehringer Ingelheim Animal Health (BIAH), 1110 Airport Pkwy, Gainesville, GA 30501, USA; 4 State Key Laboratory of Animal Nutrition, College of Animal Science and Technology, China Agricultural University, Beijing 100193, China; yuanjm@cau.edu.cn; 5 Department of Animal and Dairy Science, University of Georgia, 425 River Rd, Athens, GA 30602, USA; rrekaya@uga.edu

**Keywords:** *E. maxima*, mRNA expression, apparent ileal digestibility, amino acids’ transporters, broilers

## Abstract

**Simple Summary:**

Heat stress (HS) and *Eimeria* (*E*.) *maxima* infection are the most common physical and pathological stressors in chicken houses, and both affect intestinal digestibility and absorption leading to reduction in growth, morbidity, and mortality, causing massive economic losses. This study identifies the impact of each stressor and their combined effects on apparent amino acid digestibility and molecular transporters expression in the ileum of broiler chicken. Heat-stressed chickens showed no change in amino acids digestibility, despite the reduction in feed intake. Combining HS and *E. maxima* infection modulated the reduction in amino acids digestibility observed in the infected chickens. The expression of the ileal amino acid transporters was severely impacted by *E. maxima* infection but not by HS. Interestingly, the infected group reared under HS exhibited significantly higher expression levels in all the enterocytic apical and about half of the basolateral amino acid transporters than the infected birds raised in thermoneutral environment. Thus, HS putatively curtailed the maldigestion effects of *E. maxima*.

**Abstract:**

*Eimeria (E.) maxima* invades the midgut of chickens and destroys the intestinal mucosa, impacting nutrient digestibility and absorption. Heat stress (HS) commonly affects the broiler chicken and contributes to inflammation and oxidative stress. We examined the independent and combined effects of HS and *E. maxima* infection on apparent amino acid ileal digestibility (AID) and mRNA expression of amino acid transporters in broiler chickens (Ross 708). There were four treatment groups: thermoneutral-control (TNc) and infected (TNi), heat-stress control (HSc) and infected (HSi), six replicates of 10 birds/treatment. Ileal content and tissue were sampled at 6 d post infection to determine AID and transporters expression. Surprisingly, the HSi chickens exposed to two critical stressors exhibited normal AID. Only the TNi group displayed reduction in AID. Using TNc as control, the HSc group showed upregulated CAT1, LAT4, TAT1, SNAT1, and SNAT7. The HSi group showed upregulated CAT1 and LAT1, and downregulated b^0,+^AT, rBAT, SNAT1, and SNAT2. The TNi group showed upregulated CAT1, LAT1, and SNAT1 and downregulated B^0^AT1, b^0,+^AT, rBAT, LAT4, and TAT1. The expression of all enterocytic-apical and about half of the basolateral transporters was higher in the HSi group than in the TNi group, indicating that HS can putatively alleviate the *E. maxima* adverse effect on ileal digestion and absorption.

## 1. Introduction

Coccidiosis is the most aggressive infection caused by the *Eimeria* parasite (Phylum Apicomplexa), invading the intestine of meat-type (broiler) chickens and causing growth depression, high morbidity, and mortality [1]. *Eimeria* spp. are characterized by a high specificity of host and tissue tropism [2]. *Eimeria* (*E*.) *maxima* distinctly infects the midgut proximal and distal to Meckel’s diverticulum and along the ileum. *E. maxima* is distinguished from other *Eimeria* spp. invading chickens by generating the largest oocysts and male gamonts [3]. *E. maxima* sporozoites are released upon ingestion of sporulated oocysts. These oocysts invade the enterocytes to produce a large number of merogonic and gametogenic stages, destroying the intestinal mucosa, and severely impacting nutrients digestibility and absorption. The intestinal mucosa eventually suffers from damage and sloughing, increasing the susceptibility to secondary pathogenic invasion [4,5]. The pathogenic impact of *E. maxima* relies on multiple factors, such as the strain, the amount of ingested sporulated oocysts, and the status of host immunity and intestinal integrity [6,7].

Heat stress, caused by high ambient temperature, is a seasonal stressor in the chicken house in temperate regions and all year round in the tropical and subtropical locations. Broiler chickens respond to both acute and chronic HS by reducing feed intake and growth with worsened feed conversion ratio (FCR) [8,9]. Heat stress also causes intestinal injury and affects the immune response [10]. However, a small non-significant increase in the nutrient and amino acid digestibility was reported in HS-exposed broiler chickens [11]. Nevertheless, the expression levels of the nutrient and amino acid transporters may be modulated to compensate for the reduction in feed intake [12].

The ileum is the third portion of the small intestine, following the duodenum and jejunum, and contains a villi-rich lumen that increases the absorptive surface area. The ileum lumen is lined by highly polarized mucosal epithelial cells, mainly enterocytes, conferring a physical barrier against pathogens and controlling the transportation of nutrients, ions, and fluid [13,14]. Enterocytes express a group of transporters and binding proteins on both the apical membrane at the luminal side and basolateral membrane at the vascular side to absorb digested nutrients [15]. Amino acid absorption and transportation from the intestinal lumen to the bloodstream is a complex process and can be uni- or bi- directional across the enterocytic plasma membrane. Amino acid transporters differ in structure (i.e., monomer or dimer), solute specificity (i.e., Na^+^, K^+^, H^+^, or Cl^−^), amino acid specificity (i.e., neutral, cationic, anionic, or branched amino acids), function (i.e., uniporter, symporter, or exchanger), and physiological activity (i.e., electroneutral or electrogenic) [13].

Under different stress conditions, either physical or infectious, the enterocytes may alter nutrient transporter expression in response to stress-related molecular signals to fulfill different body requirements [16]. The nutrient digestibility and transporter expression of the intestine were previously used as functional and physiological indicators to assess gut health and mucosal integrity in response to several stressors or pathological conditions [17,18,19]. 

This study aimed to investigate apparent ileal amino acids digestibility and mRNA expression of amino acid transporters in broiler chickens infected with *E. maxima* and raised under thermoneutral or chronic heat-stress conditions.

## 2. Materials and Methods

All animal procedures in this study were conducted under the Animal Use Proposal (AUP) no. A2015 04-005, revised and approved by the Animal Care and Use Committee (IACUC) of the University of Georgia. 

### 2.1. Experimental Design and Sampling

The study had four treatments with two infection levels (infected and control) and two temperature levels (20 °C and 35 °C). The *E. maxima* sporulated oocysts were cloned from a single oocyst of a North Carolina field strain. The cloning protocol and dose formulation were described in Schneiders et al. [20]. A total of 120 two-week-old Ross 708 male broiler chickens were randomly allocated into 12 cages. The chickens were housed at two rooms with temperature levels 20 °C (TNi) or 35 °C (HSi) after being infected via gavage with 2 × 10^5^ *E. maxima* sporulated oocysts/bird suspended in distilled water. Another 120 Ross 708 male broiler chickens were randomly distributed into 12 cages in two other rooms at temperature levels 20 °C (TNc) or 35 °C (HSc), after being gavaged with distilled water as a mock-infection. Treatment groups were raised at their respective temperature continuously from 0 to 14 days post-infection (dpi). Prior to the trial, there chickens were raised on an elevated wire floored pen according to the management and husbandry standards provided by the Ross 708 (2018) manual without using any coccidia controlling program [21]. During the study, chickens were grown in wired-floor cages, supplied with *ad libitum* water, and fed on a non-medicated standard grower corn-soybean meal-based diet containing 0.2% titanium oxide. The diet analysis is described in Table S1. The *E. maxima* infection was confirmed by detecting the oocysts shed in feces of the infected groups (TNi and HSi) at 6 dpi. At 6 dpi, five birds from each treatment were randomly selected and euthanized by cervical dislocation to collect approximately one cm of ileal tissue prior to the Meckel’s diverticulum in cryovials after being rinsed with PBS and snap-frozen in liquid nitrogen. Samples were kept at −80 °C for long-term storage. The ileum content of one randomly selected bird from each cage was sampled by gently squeezed into aluminum foil plates (*n* = 6/treatment).

### 2.2. Apparent Digestibility

Ileum contents were heat-dried at 75 °C and stored at −80 °C. Dried samples were sent to the Agricultural Experiment Station Chemical Laboratories (ESCL), University of Missouri, Columbia, MO, USA for complete amino acid profiling using AOAC Official Method 982.30 E (a,b,c), chp. 45.3.05, and alkaline hydrolysis—AOAC Official Method 988.15, chp. 45.4.04, for total amino acids and Tryptophan quantification, respectively [22]. Using the reported amino acids concentrations, the AID was estimated as designed by Edwards and Gillis [23]:

$$\mathrm{AID}=100\;–\;\left[100\times \frac{\mathrm{Ti}\;\mathrm{in}\;\mathrm{diet}\;\left(\mathrm{ppm}\right)}{\mathrm{Ti}\;\mathrm{in}\;\mathrm{ileum}\;\left(\mathrm{ppm}\right)}\times \frac{\%\;\mathrm{nutrient}\;\mathrm{in}\;\mathrm{ileum}\;}{\%\;\mathrm{nutrient}\;\mathrm{in}\;\mathrm{diet}}\right]$$

### 2.3. Nucleic Acid Extraction

The RNA was extracted from 100 mg of each frozen ileal tissue sample by Trizol-Chloroform method [24].

### 2.4. Gene Expression

A NanoDrop 2000 Spectrophotometer (Thermo Fischer Scientific, Waltham, MA, USA) was used to measure the RNA concentration in the purified samples. All samples were diluted with nuclease-free water to RNA concentration of 200 ng/µL. High-Capacity cDNA Reverse Transcription Kit (Thermo Fischer Scientific, Waltham, MA, USA) master mix was added to 10 µL of each diluted RNA sample, according to the manufacturer’s protocol. To generate cDNA, the mixture was placed in the Gradient Mastercycler (Eppendorf, Framingham, MA, USA) adjusted as: 10 min at 25 °C, 120 min at 37 °C, 5 min at 85 °C, and the end cycle at 4 °C. Obtained cDNA was stored at −25 °C. The cDNA samples’ concentrations were measured using NanoDrop 2000 Spectrophotometer, and each was diluted to 20 ng/µL prior to quantification. The quantitative analysis was performed with RT-qPCR in triplicates by adding 2 µL of diluted cDNA (20 ng/µL), 0.3 µL of each primer (forward and reverse, each of concentration 10 µM) (Table 1), 7.4 µL of nuclease-free deionized water, and 10 µL of Fast SYBR^TM^ Green Master Mix^®^ (Applied Biosystems, Waltham, CA, USA). The StepOnePlus machine (Applied Biosystems, Waltham, MA, USA) was used for the RT-qPCR analysis and adjusted to cycles: 50 °C for 120, and 95 °C for 120 s, followed by 40 cycles of 95 °C for 15 s, and 60 °C for 60 s, with reporting the Ct values at the end of each cycle and the melting temperature curve. The relative gene expression of the investigated genes was normalized against the expression of β-actin (endogenous control), and then, the expression fold increase was determined versus the control group (TNc). The results were calculated as 2^−∆∆*ct*^ [25] for the following amino acid transporters of solute carrier family (SLC): B^0^AT (SLC6A19), b^0,+^AT (SLC7A9), rBAT (SLC3A1), TAT1 (SLC16A10), LAT4 (SLC43A2), CAT1 (SLC7A1), LAT1 (SLC7A5), SNAT1 (SLC38A1), SNAT2 (SLC38A2), and SNAT7 (SLC38A7).

### 2.5. Statistical Analysis

A two-way ANOVA was implemented using the SAS^®^ Studio software (SAS Institute Inc., Cary, NC, USA). The model investigates the differences in AID of amino acid and the ileal expression of the amino acid transporters between the treatment groups. The model was performed using the generalized linear model (GLM) procedure (SAS, 2018) [26]. Multiple comparison between treatment groups were implemented using the “Tukey–Kramer” option. The difference was considered significant at *p* < 0.05. 

## 3. Results

**Figure 1:**
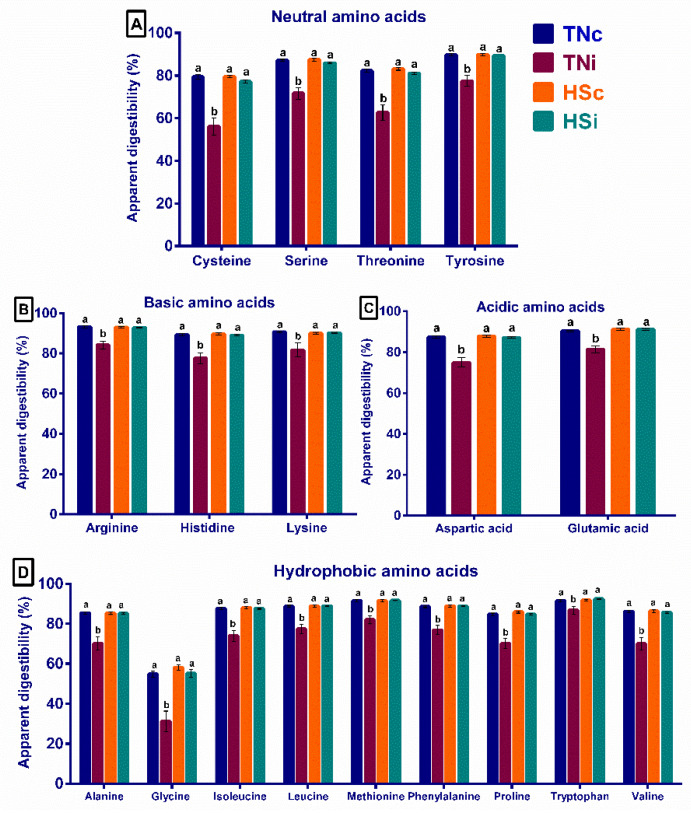
The apparent ileal digestibility of the neutral (**A**), basic (**B**), acidic (**C**), and hydrophobic (**D**) amino acids at 6 dpi of *E. maxima* infected chickens raised under thermoneutral (TNi) and heat-stress (HSi) conditions, as compared to uninfected control chickens raised under thermoneutral (TNc) or heat-stress (HSc) conditions (N = 6). Significant differences (*p* < 0.05) are depicted by different letters. The error bars represent the SEM.

The AID of all investigated proteinogenic amino acids (cysteine, serine, threonine, tyrosine, arginine, histidine, lysine, aspartic acid, glutamic acid, alanine, glycine, isoleucine, leucine, methionine, phenylalanine, proline, tryptophan, and valine) at 6 dpi significantly decreased by 20–40% in the TNi group, contrasted with the other treatment groups, and there were no differences between the TNc, HSc, and HSi groups (Figure 1A–D).

**Figure 2:**
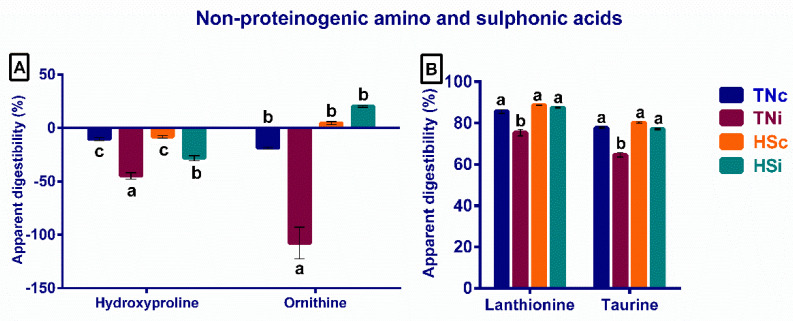
The apparent ileal digestibility of the non-proteinogenic amino acids (**A**) and sulphonic acids (**B**) at 6 dpi of *E. maxima*-infected chickens raised under thermoneutral (TNi) and heat-stress (HSi) conditions, as compared to uninfected control chickens raised under thermoneutral (TNc) or heat-stress (HSc) conditions (N = 6). Significant differences (*p* < 0.05) are depicted by different letters. The error bars represent the SEM.

The non-proteinogenic amino (hydroxyproline and ornithine) and sulphonic acids (lanthionine and taurine) displayed similar AID pattern, even though the AID of the hydroxyproline and ornithine were negative (Figure 2A,B). The TNi group showed the least AID of lanthionine and taurine compared with the other treatment groups. Furthermore, the TNi group had the highest ileal concentration of hydroxyproline and ornithine compared with the other treatment groups (Figure 2A,B).

**Figure 3:**
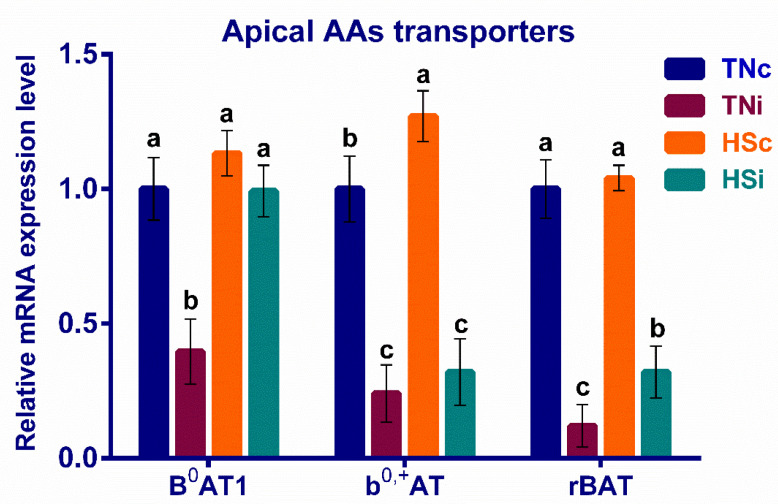
The mRNA fold expression of the ileal apical amino acid transporters at 6 dpi of *E. maxima* infected chickens raised under thermoneutral (TNi) and heat-stress (HSi) conditions, as compared to uninfected control chickens raised under thermoneutral (TNc) or heat-stress (HSc) conditions (N = 5). Assays were run in triplicate. RT-qPCR expression results were depicted as 2^−ΔΔCt^. Significant differences (*p* < 0.05) are depicted by different letters. The error bars represent the SEM.

**Figure 4:**
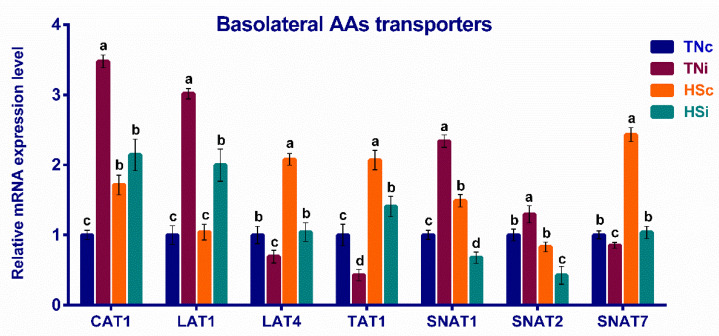
The mRNA fold expression of the ileal basolateral amino acid transporters at 6 dpi of *E. maxima* infected chickens raised under thermoneutral (TNi) and heat-stress (HSi) conditions, as compared to uninfected control chickens raised under thermoneutral (TNc) or heat-stress (HSc) conditions (N = 5). Assays were run in triplicate. RT-qPCR expression results were depicted as 2^−ΔΔCt^. Significant differences (*p* < 0.05) are depicted by different letters. The error bars represent the SEM.

The mRNA expression of the enterocytic apical and basolateral amino acids are presented in Figure 3 and Figure 4, respectively. The TNi group exhibited the lowest mRNA expression levels of the enterocytic apical amino acid transporters B^0^AT, b^0,+^AT, and rBAT and the basolateral transporters TAT1, LAT4, and SNAT7, compared with the other treatment groups. However, the difference between the b^0,+^AT expression levels in the TNi and HSi chickens was not significant (*p* = 0.067). The TNi group exhibited the highest expression levels of the basolateral amino acids’ transporters CAT1, LAT1, SNAT1, and SNAT2, compared with the other treatment groups. The b^0,+^AT, CAT1, LAT4, TAT1, SNAT1, and SNAT7 transporters were upregulated in the HSc group contrasted with the TNc group (Figure 3). The B^0^AT, b^0,+^AT, rBAT, LAT4, TAT1, and SNAT7 transporters’ expression levels were downregulated, while CAT1, LAT1, SNAT1, and SNAT2 were upwardly expressed in the TNi group compared with the HSi group (Figure 4).

## 4. Discussion

Broiler chickens exposed to HS reduce feed consumption in order to decrease internal heat generation [27,28]. *E. maxima* infection has been shown to impact the growth and feed consumption of the chickens due to intestinal injury and related complications [29,30]. It is reported that the impact of *E. maxima* infection on growth and FCR was higher than that of HS at 7 dpi [24].

### 4.1. The Effect of HS and E. maxima Infection on the Amino Acid AID

*Eimeria* spp. are intestinal parasites that exclusively infect and devastate the mucosal cells resulting in a massive depression in nutrient digestibility and absorption [17,31]. On the other hand, chickens subjected to HS manifested intestinal leakage [27] with a normal digestibility profile [12,16]. The current study also observed that amino acid AID values were significantly reduced only in the TNi group but was normal in the HSc contrasted with the TNc group (Figure 1A–D), which is similar to data reported by Habashy et al. [12]. Interestingly, HSi group showed normal amino acid AID values compared with the TNc group (Figure 1A–D), suggesting that HS alleviated the negative impact of *E. maxima* infection on the AID observed in the TNi group. 

All the investigated amino acids are proteinogenic except hydroxyproline, ornithine, and lanthionine, which are non-proteogenic. The non-proteinogenic amino acids do not possess codon triplets or specific tRNA. They are metabolites, or intermediates in biosynthesis, or produced during the post-transitional modification of the proteinogenic amino acids [32,33]. The AID of hydroxyproline and ornithine were negative because their concentration in the ileum content was higher than that in feed. Interestingly, the TNi group significantly expressed the highest ileal hydroxyproline and ornithine concentrations (Figure 2A,B). Both amino acids, hydroxyproline and ornithine, are involved in the proline cycle in the mammalian intestine but not in the chicken intestine due to the lack of proline-5-carboxylate enzyme [34,35]. They are highly excreted in the injured tissue exudate [36,37]. Further, those two amino acids are intermediates in the biosynthesis of the extracellular matrix (mainly collagen) and contribute to wound healing [38,39]. This suggests that the TNi group may have suffered a significantly higher degree of intestinal tissue injury than the other treatment groups.

### 4.2. The Effect of HS and E. maxima Infection on the Apical Amino Acid Transporters

Enterocytes, as polarized cells, express specific amino acid transporters on their apical membrane, including B^0^AT1, b^0,+^AT, and rBAT, which are rarely expressed on other non-polarized cells [14,15]. The amino acid transporter B^0^AT1 is a Na^+^-dependent neutral amino acid symporter of system B^0^ that mediates the absorption of all neutral hydrophilic and hydrophobic amino acids [40,41]. The B^0^AT1 protein mediates the absorption of almost all the neutral amino acids, including eight essential amino acids (glycine, leucine, isoleucine, methionine, phenylalanine, threonine, tryptophan, and valine) that act as building blocks for protein synthesis. These essential amino acids are also involved in metabolic and immune functions [42,43]. The enterocytes exhibit a sufficient expression level of B^0^AT1 under HS putatively to maintain a constant absorption of the glutathione precursors (i.e., cysteine and glycine) [44,45].

The b^0,+^AT is a light subunit that heterodimerizes with a heavy subunit (rBAT) to become a heteromeric transporter (Figure 3) [46]. The b^0,+^AT/rBAT complex is a broad-specificity cationic- and neutral-amino-acid antiporter of system b^0,+^ with a y^+^L-like activity that controls the uptake of the cationic (basic) amino acids (arginine, histidine, lysine) in exchange for neutral amino acids [40,47]. Exposing broiler chickens to chronic HS resulted in the downregulation of B^0^AT and the normal expression of b^0,+^AT [12]. The current study showed normal expression of B^0^AT1 and rBAT and the upregulation of b^0,+^AT in the HSc group compared with TNc group (Figure 3). The inconsistency between the two studies could result from the difference in experimental design (e.g., intensity and duration of HS). Previous studies, as well as the current study, showed that all the apical transporters B^0^AT1, b^0,+^AT, and rBAT were significantly downregulated in the *E. maxima*-infected chickens under thermoneutral conditions (TNi) compared with the TNc group (Figure 3) [29,30,48], possibly resulting in the reduction in growth associated with *E. maxima* infection. The HSi group showed the downregulation of b^0,+^AT, and rBAT, while B^0^AT1 is normally expressed contrasted with the TNc group (Figure 3). Interestingly, the HSi group showed higher expression levels of the three apical transporters than the TNi group (Figure 3). Schneiders et al. [24] reported that HS curtails the sexual stage of *E. maxima* life cycle. Taken together, the apical amino acid transporters’ expression difference and the potential reduction in sexual stages of *E. maxima* under HS could explain the improved digestibility in the HSi group, compared with the TNi group. 

### 4.3. The Effect of HS and E. maxima Infection on the Basolateral Amino Acid Transporters

The mRNA expression levels of the enterocytic basolateral amino acid transporters CAT1, LAT1, LAT4, TAT1, SNAT1, SNAT2, and SNAT4 were also investigated [14]. CAT1 is a cationic amino acid uniporter of system y+ that maintains about an eight-fold inward concentration gradient of cationic amino acids across the plasma membrane [49]. In concordance with the current study (Figure 4), upregulated ileal CAT1 transporter was reported in the *E. maxima* infected broilers [30,48].

LAT1 is a light catalytic subunit of system L that dimerizes with a heavy glycoprotein subunit (4F2hc) to form a Na^+^-independent large neutral and aromatic amino acids exchanger. The LAT1/4F2hc exchange mechanism enables the relative concentration equilibrium of the large neutral amino acids across the plasma membrane [50,51]. LAT1 upregulation was observed in T lymphocytes upon IL-2 stimulation or antigenic activation. This suggests that the T lymphocyte activation may have contributed to the LAT1 upregulation observed in the infected groups (Figure 4). It was thought that the pathogen-induced system L expression (LAT1) provides intracellular leucine supply required for the activation of Mammalian Target of Rapamycin Complex 1 (mTORC1) that forms a serine/threonine kinase complex essential for T lymphocyte activation, proliferation, and differentiation [52].

LAT4 is a Na^+^-independent, neutral amino acids uniporter of system L that exhibits a wide tissue distribution. The intestinal LAT4 is mainly distributed in the crypt cells indicating low contribution to the net amino acid absorption [53,54]. The LAT4 gene was upregulated in the embryonic mouse hypothalamic cell line N25/2 in response to amino acids starvation [55]. The amino acid starvation-induced by HS could putatively contribute towards the upregulation of LAT4. The study by Habashy et al. [12] reported similar results. Expectedly, the ileal LAT4 expression was impacted by *E. maxima*-induced mucosal damage in the infected groups. However, the HSi group expressed normal LAT4 expression, suggesting that HS may have restored the LAT4 expression when combined with *E. maxima* infection (Figure 4).

TAT1 is an aromatic amino acids uniporter of the T system, H^+^ monocarboxylate transporter family. Unlike LAT4, the intestinal expression of TAT1 is mostly localized toward the villi tips [56,57]. TAT1 is the principal route for efflux of the aromatic amino acids that entered the cell through the heterodimeric exchanger LAT2/4F2hc exchanging for small neutral amino acids [58]. TAT1 is also responsible for lactic acid efflux and influx across the cell membrane [59]. The regulation of TAT1 expression has been reported in the skeletal muscle tissue of humans and rats but not in enterocytes [60,61]. The activation of AMP-activated protein kinase (AMPK) upon the accumulation of AMP during exercise was suggested to mediate the upregulation of TAT1 [61]. Since the AMP accumulation and AMPK activation in various body tissues were associated with stress response, including HS, we can hypothesize that a similar mechanism may have contributed to the upregulation of TAT1 in the HS groups (HSc and HSi) [12,62].

SNAT1 and 2 are Na^+^-dependent neutral amino acids symporters of system A with broad specificity and high affinity to glutamine [63]. Glutamine is an essential amino acid for the activity of the central regulator of the immune cells promoting mTORC1 activation. The CD3 and CD28 activated T-cells exhibited elevated expression levels of SNAT1 and SNAT2 [64,65]. Therefore, potentially increasing the activated T-cells number in the ileum of the TNi group may contribute to the observed SNAT1 and SNAT2 upregulation (Figure 4).

SNAT7 is a Na^+^-dependent neutral amino acids symporter/H^+^-antiporter of system N with system A-like wide substrate specificity [66]. SNAT7 was detected on the lysosomal membranes [67,68]. The upward expression of SNAT7 in the HSc group compared to other treatment groups may be associated with the profuse lysosomal activity. It has been documented that the lysosomal system can terminate the cellular oxidizing elements resulting from HS-associated oxidative stress [69].

## 5. Conclusions

The current study shows that *E. maxima* infection under thermoneutral condition affected amino acid digestibility. Chickens exposed to HS alone or combined with *E. maxima* infection had normal apparent amino acid digestibility. Increased hydroxyproline and ornithine concentration only in the TNi group indicates higher tissue damage as compared with the other treatment groups. Furthermore, the amino acid transporter expression levels of the HSi group did not change significantly compared with the other groups. Taken together, the current study suggests that HS limits the pathogenic effect of *E. maxima* on ileum enterocytes, including the digestibility and amino acid transporters expression. The data also indicate that the amino acids and their transporters could play multiple molecular functions upon the physiological and pathological status of the enterocyte, in addition to their primary nutritional role. Amino acid dynamics could pave the way for nutritional recommendations that can alleviate the effect of HS and/or *E. maxima* infection in broiler chickens.

## Figures and Tables

**Table 1:** Primer used to detect the expression levels of the amino acids’ transporters using RT-qPCR.

Nutrient Transporter	Transporter Gene	Gene Bank Accession Number	Size	Align.	Primers Sequences
Apical amino acids	B^0^AT1 (SLC6A19)	XM_419056.8	117	Forward	5′CTGCCTGGGTTTGTCATCTAT3′
				Reverse	5′GCGCAGACGATACCTGTAAT3′
	b^0,+^AT (SLC7A9)	NM_001199133.1	113	Forward	5′GATCCCTGGAGCCTGAATTAC3′
				Reverse	5′CTCCTTTCTGTTGTCCTGTTCT3′
	rBAT (SLC3A1)	XM_004935370.3	119	Forward	5′CTGAGAGCATCACAGCCTATTC3′
				Reverse	5′GCCAGGTTCACTGCTGTATT3′
Basolateral amino acids	TAT1 (SLC16A10)	NM_001321736.1	119	Forward	5′GCACCATCGAACCTCTGTATT3′
				Reverse	5′CACTAGACCAAGGCGTTTCTT3′
	LAT4 (SLC43A2)	XM_415803.6	113	Forward	5′GACTCGCAGCATCCCTAAAT3′
				Reverse	5′GTGTCAGAGAAGTGGACGATATG3′
	CAT1 (SLC7A1)	NM_001145490.1	111	Forward	5′CGAACAACAGAGGAGACAGATAA3′
				Reverse	5′GGGACACAGTATGGCTTTGA3′
	LAT1 (SLC7A5)	NM_001030579.2	98	Forward	5′GCCTTCTCCAATGACATCTTCT3′
				Reverse	5′TAACGCAGCCACATCATACC3′
	SNAT1 (SLC38A1)	NM_001199603.1	108	Forward	5′CGCTAAATGCAACATCACCTATC3′
				Reverse	5′TGGTGGGCAAAGCATACA3′
	SNAT2 (SLC38A2)	NM_001305439.1	127	Forward	5′GAACAAGTAGGGCCCTGTAATC3′
				Reverse	5′GGGCAGAGCTTGATGTTATCT3′
	SNAT7 (SLC38A7)	XM_025154307.1	93	Forward	5′CAAGTTCACCATCAGCATCAC3′
				Reverse	5′CTCAGAGAGCTGGCGTATTT3′
	B-actin	NM_205518.2	125	Forward	5′AGACATCAGGGTGTGATGGTTGGT3′
				Reverse	5′TCCCAGTTGGTGACAATACCGTGT3′

## Data Availability

The data presented in this study are available on request from the corresponding author.

## Supplementary Materials

The following supporting information can be downloaded at: https://www.mdpi.com/article/10.3390/ani12121554/s1, Table S1: The feed analysis.
