# Supplementary material for: Heat Stress Alters the Effect of Eimeria maxima Infection on Ileal Amino Acids Digestibility and Transporters Expression in Meat-Type Chickens
Source: Animals (Basel). 2022 Jun 16;12(12):1554. doi: 10.3390/ani12121554 (PMC9219439; doi:10.3390/ani12121554)
Supplement: Supplementary file 1 [file animals-12-01554-s001.zip › animals-1724506-supplementary.pdf]

Supplementary Table

Table S1. The feed analysis.

Element	%
Taurine	0.19
Hydroxyproline	0.03
Asparticacid	2.05
Threonine	0.78
Serine	0.88
Glutamicacid	3.57
Proline	1.09
Lanthionine	0.02
Glycine	0.84
Alanine	0.99
Cysteine	0.32
Valine	1.00
Methionine	0.43
Isoleucine	0.91
Leucine	1.68
Tyrosine	0.64
Phenylalanine	1.02
Hydroxylysine	0.03
Ornithine	0.02
Lysine	1.16
Histidine	0.54
Arginine	1.32
Tryptophan	0.26
Grossenergy(Kcal/100g)	362.80
Crudeprotein	18.76
Moisture	11.02
Crudefat	5.64
Crudefiber	2.63
Ash	5.33
